# Fungal decomposition of terrestrial organic matter accelerated Early Jurassic climate warming

**DOI:** 10.1038/srep31930

**Published:** 2016-08-24

**Authors:** Grzegorz Pieńkowski, Marta Hodbod, Clemens V. Ullmann

**Affiliations:** 1 Polish Geological Institute – National Research Institute, Rakowiecka 4, PL-00-975 Warszawa, Poland; 2 University of Exeter, Camborne School of Mines and Environment and Sustainability Institute, Penryn Campus, Penryn, Treliever Road, TE10 9FE, UK

## Abstract

Soils – constituting the largest terrestrial carbon pool - are vulnerable to climatic warming. Currently existing uncertainties regarding carbon fluxes within terrestrial systems can be addressed by studies of past carbon cycle dynamics and related climate change recorded in sedimentary successions. Here we show an example from the Early Jurassic (early Toarcian, c. 183 mya) marginal-marine strata from Poland, tracking the hinterland response to climatic changes through a super-greenhouse event. In contrast to anoxia-related enhanced carbon storage in coeval open marine environments, Total Organic Carbon (TOC) concentrations in the Polish successions are substantially reduced during this event. Increasing temperature favoured fungal-mediated decomposition of plant litter – specifically of normally resistant woody tissues. The associated injection of oxidized organic matter into the atmosphere corresponds to abrupt changes in standing vegetation and may have contributed significantly to the amplified greenhouse climate on Earth. The characteristic Toarcian signature of multiple warm pulses coinciding with rapidly decreasing carbon isotope ratios may in part be the result of a radical reduction of the terrestrial carbon pool as a response to climate change.

The terrestrial carbon pool and carbon cycle are characterized by complex, non-linear behaviour^1^,^2^,^3^,^4^ and is consequently hard to model robustly^5^. A very narrow time span of the instrumental record adds substantial uncertainties to the understanding of the system’s behaviour during recent anthropogenic climate change. This information gap can be reduced using data from past climate analogues, which can extend our knowledge of causal links between different mechanisms of climate warming events. The Early Jurassic Toarcian Oceanic Anoxic Event (T-OAE, beginning at c. 183.22 mya and lasting for some 300 kya^6^,^7^ or 500–900 kya^8^, was one of the most marked global warming events (Fig. 1A) of the geologic past. Severe climate change and biological turnover of the Early Toarcian can thus serve as a model for fast anthropogenic climate change, which is predicted to turn the terrestrial biosphere from an overall carbon sink into a source by about 2050^1^,^9^.

The T-OAE is associated with profound disturbances in geochemical, sedimentary, and paleontological records^10^,^11^,^12^,^13^,^14^,^15^ and characterized by a succession of −4 to −8‰ stepped carbon-isotope excursions (CIEs) recorded globally in marine and continental carbonates and organic matter^10^,^11^,^16^,^17^,^18^. It is currently believed that this event was triggered by large-scale volcanic eruptions, preceded by the sill emplacement and magma outgassing in the Karoo-Ferrar basaltic province^6^,^19^, causing a rapid increase in atmospheric pCO_2_ from 350+/−100 p.p.m.v to 1200+/−400 p.p.m.v^20^ and average air temperatures (ca. +5°C)^14^,^21^. This injection of greenhouse gas into the atmosphere is further believed to have facilitated pulsed, astronomically paced releases of methane hydrate along continental margins, induced widespread marine anoxia^11^,^15^ and led to marine accumulations in organic matter as evidenced by widespread, black bituminous shales^7^,^10^,^11^.

Environmental disturbances preconditioning the biosphere for this major event have been identified, notably the Pliensbachian–Toarcian boundary (Pl/To) c. 184 mya ago^17^,^21^. This boundary event was connected with a marine regression/transgression couplet, ‘precursor’ CIE^17^,^18^,^21^, onset of a global extinction^22^ and intermittent global warming. While the anticipated triggers for the Pl/To event are similar to those invoked for the T-OAE, the Pl/To boundary event is believed to have had less severe environmental impact^23^. Compared with the Early Toarcian CIEs, the precursor CIE occurred in a transitional context from supposed icehouse to greenhouse conditions^14^.

While numerous studies of Lower Toarcian strata for the marine realm exist, which address oceanographic, climatic and biological processes that drove the Earth’s system into the T-OAE, investigations regarding climatic trends in the continental realm (e.g., ref. 18) remain sparse. To better understand terrestrial responses to the T-OAE, the present study employs geochemical and palynological information from sedimentary archives of the Polish Basin.

The Polish marginal-marine strata coeval to the T-OAE carbon rich, marine black shales (Fig. 1) are represented by poorly consolidated green/grey mudstones, claystones and siltstones with subordinate sandstone intercalations basin (Ciechocinek Formation), formed in a shallow (max. ~14–30 m water depth), large brackish marine embayment/lagoon fringed by a coastal-deltaic environment, located at the Toarcian times around 40–45°N^23^ (Fig. 1). Most of the mineral grains in the Ciechocinek Formation are of detrital origin and sourced from strongly weathered metamorphic and sedimentary rocks^24^,^25^. Early diagenetic phases include Fe-rich chlorite, as well as siderite and pyrite, which are genetically linked with the brackish-marine environment. The sediments were deposited and were studied in detail in four fully-cored borehole profiles – Figs 1 and 2. Based on carbon isotope stratigraphy, using fossil wood and recording the series of stratigraphically abrupt 5 CIE steps characteristic of the T-OAE, the Ciechocinek Formation strata were correlated to marine strata from England^16^,^18^,^26^. These CIEs are most prominently visible in the expanded records of Mechowo, Gorzow Wielkopolski and Brody-Lubienia (Fig. 2). Sea-level changes, well expressed in correlatable shallowing-upward parasequences, basin, are coeval with carbon isotope shifts, with some discrepancies in the upper cycles caused by intensification of weathering and runoff^18^. During the T-OAE humid conditions (with perennially high humidity and rapid weathering) and enhanced hydrological cycle resulted in enhanced erosion associated with more sandy facies^23^ (Fig. 2) and also in elevated kaolinite content^27^.

## Results

Organic matter (=kerogen, palynological matter, palynomacerals^28^ in the Ciechocinek Formation is entirely represented by the type III kerogen (most commonly, type IV inertinite^29^) of terrestrial (plant) origin^30^. It is characterized by low thermal maturity and low Hydrogen Index (HI index below 200 – except for one sample, usually below 100) (Supplementary Tables 1–2). While the kerogen type remains largely the same throughout the studied interval, environmental/climatic changes in the hinterland lead to strongly differing TOC content and carbon isotope ratios (Fig. 2). Additionally, during the T-OAE, the Oxygen Index (OI) is by an order of magnitude higher than before and after this interval (Fig. 2, Supplementary Table 1), and shows good correlation with CIE steps (Fig. 2).

TOC content and carbon isotope ratios (Supplementary Table 1; Fig. 2) in the strata coeval to T-OAE are strongly depleted despite growing proximity of the coastal vegetation zone. This depletion in TOC and δ^13^C causes two populations of data in a cross plot of TOC versus δ^13^C (Fig. 3c). One population has δ^13^C>−25.5‰ and generally high TOC (median = 1.1%) and represents background sedimentation whereas the other one has δ^13^C<−25.5‰ and low TOC (median = 0.3%) and represents the event beds. These two populations demonstrate that there is a systematic difference between the TOC preservation during periods of very negative δ^13^C and episodes with less negative δ13C. This separation is especially clear in the Mechowo data (Fig. 3c; Supplementary Table 1).

**Figure 1:**
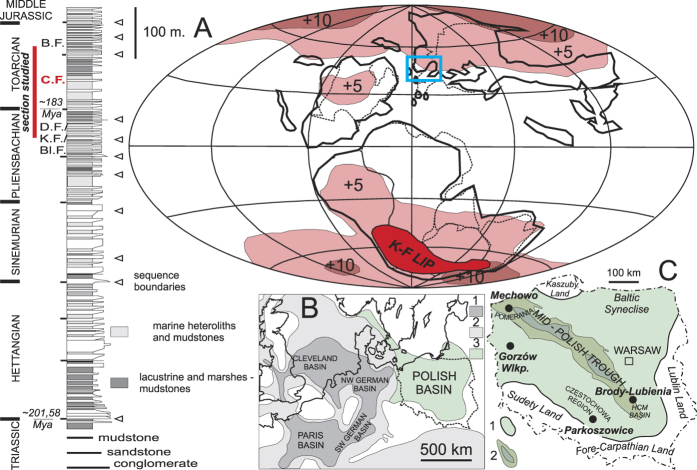
Study area and generalised profile of the Lower Jurassic in Poland. Stratigraphy: D.F. – Drzewica Formation; K.F. – Komorowo Formation; Bl.F. – Blanowice Formation; C.F. – Ciechocinek Formation; B.F. – Borucice Formation (after 23). (**A**) simplified palaeogeographic map of the Earth during the T-OAE: continents and air temperature rise based on simulation of global paleoclimatic changes during the Early Toarcian warming event (modified from 14), K-F LIP – approximate extent of the Karoo-Ferrar Large Ingeous Province (after 57); (**B**) palaeogeographic map of Europe, general Lower Toarcian lithofacies: 1. black marine shales (after 17); 2 – shallow marine deposits (after 17); 3. green shales; (**C**) Polish basin with 1. maximum extent of the Lower Toarcian deposits and 2. maximum thickness zone along the Mid-Polish Trough, and locality of boreholes studied herein (simplified after 23). Maps generated with CorelDRAW X3 v. 13.0.0.576, http://www.corel.com/pl/.

**Figure 2:**
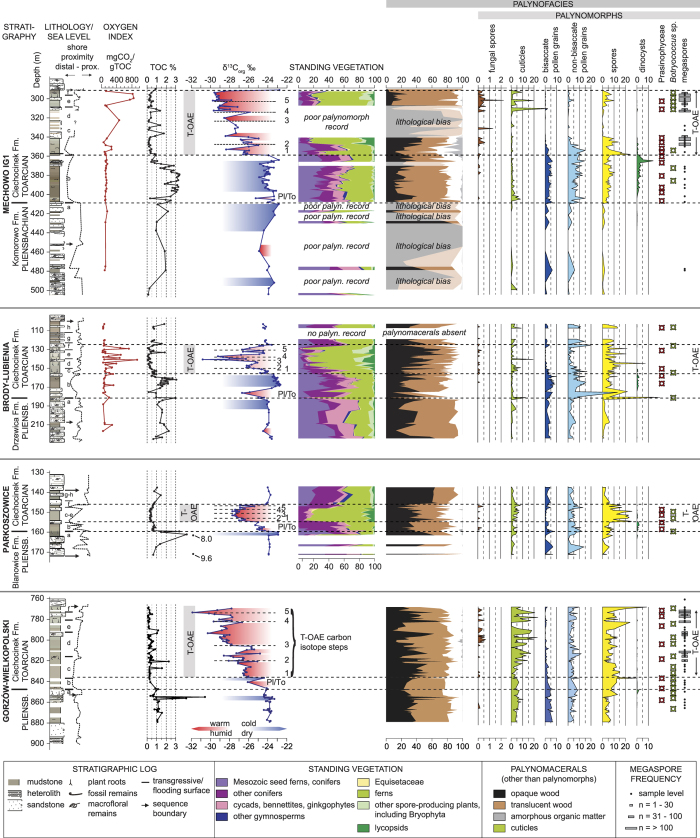
Lithological, geochemical and floral reaction to the Early Toarcian warming event (T-OAE – Toarcian Oceanic Anoxic Event). Lithological column, sea-level curve and stratigraphy after ref. 23; OI - Oxygen Index in mg CO2/gTOC; carbon isotope curve after ref. 18, climate changes marked in red and blue; standing vegetation (in three profiles) in percent of determined spore and pollen taxa; palynofacies (phytoclasts and palynomorphs) in percent of total palynodebris and palynomorphs; frequency of megaspores in two boreholes after 33,34 showed separately. Standing vegetation responds synchronous to fluctuations δ^13^C, OI, wood and cuticle content and fungal spore frequency, pointing to decomposition of terrestrial carbon pool during the warming event (T-OAE)^58^.

In the Polish Basin, the majority of kerogen is represented by wood, both translucent and opaque (Supplementary Table 2). Shapes and reflectance of opaque phytoclasts do not indicate the presence of charcoal-enriched samples. Cuticle and other plant tissues contribute usually below 10% and rarely exceed 20%, and amorphous organic matter occurs in larger (up to 20%) concentrations only in a short interval in Mechowo IG-1. Marine palynomorphs are very subordinate. A single noticeable peak of a dinoflagellate bloom (Fig. 2, Mechowo borehole) reflecting an intermittently high runoff and nutrient supply (indicated also by increase in phosphorus content^27^ and slightly preceding the onset of T-OAE, can be correlated with the marine plankton bloom heralding the T-OAE in the Paris Basin^26^,^31^. The aggregate content of land palynomorphs (spores and pollen grains) in kerogen does usually not exceed 10–20%. Fluctuations between spores and non-bisaccate pollen grains on one side and bisaccate pollen grains^32^ on the other side reflect climate changes and to lesser extent shore proximity, as the Polish Basin was a proximal setting throughout the studied interval^23^ (Fig. 1). Taphonomic bias of the palynological record can influence ecosystem- and climate reconstructions, altering the original palynomorph associations. However, these taphonomic problems are likely negligible because of tectonic stability^23^, uniformly high sedimentation rate and proximity of the coast, reducing transport bias^23^,^30^.

Independently counted megaspores show abundance peaks coincident with the development of the overall negative isotope excursion of the T-OAE. The highest frequencies of megaspores are hereby observed in the four highest sedimentary parasequences (‘c’-‘f’) which correspond to CIEs 2 to 5 in Fig. 2. The megaspores, such as the most common *Paxillitriletes phyllicus* (Murray) Hall et Nicolson 1973, and *Minerisporites institus* Marcinkiewicz, 1971^33^,^34^ are derived from the hydrophilic plant groups Lycopsida (club mosses) and Isoetaceae (quill worts)^18^,^33^,^34^, which point to very humid conditions during almost the entire T-OAE, upwards from CIE step 2 of the carbon isotope curves. Similarly humid conditions occurred in the adjacent eastern Danish basin^20^.

An approximate measure of floral species richness can be derived from the recorded spore/pollen spectrum^35^. This relationship is thought to be robust for the Jurassic floral assemblages that are less diversified than the modern counterparts. Palynodiagrams (Supplementary Table 2, Fig. 2) of three selected borehole sections reflect this standing vegetation assemblage and document the plants’ response to the Early Toarcian environmental perturbation. Palynofacies and palynodiagrams show that with each T-OAE related CIE (Fig. 2), plant assemblages indicative of hot and humid conditions increased in importance. After CIE step 4 stabilization and gradual return to pre-excursion conditions (step 5 and higher) is documented. Most negative δ^13^C values (corresponding to higher temperature and increased pCO_2_, reflected by stomatal index measured in the Danish basin^20^) are associated with frequency spikes of fern spores, which in the studied succession are mostly attributed to the Dicksoniaceae and Cyatheaceae families that are recently known as tropical plants^36^. Conifers and other pollen-producing plants indicative of relatively colder climates on the other hand were reduced during this interval. Equisetaceae, bryophyte and lycopsids point to enhanced humidity, but they are poorly related to the temperature which is more reliably shown by the ratio between fern spores and conifers/other pollen-producing plants (Figs 2 and 4).

Within the group of accessory palynomorphs, the appearance of fungal spores during the most negative peaks of the CIEs is of particular importance despite of problems in relating fungal spores to specific types of fungi known from coal macerals^37^ in palynomacerals. Observed fungal spores represent various morphotypes (Fig. 5a–e): amerospores - unicellular, aseptae, sphaerical or sack-shaped spores with a highly variable size; phragmospores - spores with transverse septa, and dictyospores - multicellate spores^38^,^39^,^40^. Superficially, some sphaerical fungal spores can resemble other palynomorphs – i.e. sphaerical Prasinophyceae. However, the latter are characterized by scabrate texture and have a thick wall Fig. 5f). Additionally, in some cases sphaerical fungal spores show structures related to hyphae attachments (Fig. 5).

The highest fungal spore abundance is associated with an increase of the cuticle fraction on the expense of wood (Supplementary Table 2; Figs 2 and 4). This increase cannot directly be compared with a change in vegetation, because cuticle was produced by most of the registered plant groups and is therefore not sensitive to changes in floral assemblages.

## Discussion

### Fidelity of Carbon isotope steps in the Polish Basin

δ^13^C of bulk organic matter in marine and marginally marine succession strongly depends on the ratio of marine versus terrestrial OM, as shown specifically for T-OAE in marine sediments in England and Germany^41^,^42^. Organic matter mixing can thus obscure changes of the isotopic composition of contemporaneous atmospheric carbon dioxide recorded in detrital remains of the standing vegetation (cf. 43). These complications of organic matter mixing can be ruled out for the Polish strata, however, because here the δ^13^C record^18^ (Fig. 2) is derived exclusively from terrestrial OM (separated wood fraction).

Biases in the wood record itself could arise, if variable carbon isotope fractionation occurred within the wood fraction. Wood material studied here is derived entirely from C3 plants (C4 plants only arose much later, in the Oligocene epoch^44^). Moreover, the wood fraction in kerogen was mostly produced by conifer trees^45^. Ferns, like other plants (lycopsids, cycads, bennettites and ginkgophytes) were generally less important wood producers at this time. Thus, differences in isotopic fractionation between conifers and other plants can be considered as insignificant. Carbon isotope fractionation factors of conifers are known to respond little to changing environmental parameters^46^. Humidity and precipitation rates can affect carbon isotope fractionation in plant materials^47^, whereby δ^13^C values are negatively correlated with mean annual precipitation in extant C3 plants. Differences in fractionation are highly significant (over 2‰) when the most contrasting plant habitats are compared - dry (below 600 mm/year) and very humid (over 2000 mm/year) precipitation rate. Constraints on the Toarcian environment in Poland coeval to the T-OAE and based on reconstruction of hydrological cycle, standing vegetation and clay mineral analysis^18^,^23^,^27^,^32^ make the presence of dry or semi-dry habitats unlikely (see also ref. 20). Overall it can be inferred that the precipitation during the T-OAE interval was not lower than ~1000 mm/year (the values of 1000mm/year are inferred from numerical a GCM model; they correspond to “annual” precipitation rates at paleolatitudes of 40°N–14). In conclusion, humidity-related fractionation effects could only slightly modulate the observed C isotope trends, rather than being their most defining factor.

Further support for CIE steps representing rapid change of δ^13^C in atmospheric CO_2_ during the T-OAE rather than a mixing phenomenon comes from other sections for which purely terrestrial organic matter has been analyzed^17^,^48^,^49^.

Additionally, negating the genuine nature of the first two CIE steps^42^ would modify correlations between terrestrially and marine-dominated successions (England and Poland in particular^18^, their Fig. 1). When following^42^ and discarding the lowermost two steps (1 and 2) of T-OAE in England, the whole T-OAE - related negative CIE observed in Poland should be correlated with the higher part of the English profile (corresponding to the steps 3, 4 and two higher ones, shown by ref. 18 – their Fig. 1). Overall, OM mixing or significantly enhanced δ^13^C fractionation in wood due to varying regional humidity as well as artificial CIE steps in the Toarcian carbon isotope stratigraphy^42^ are therefore in contrast with the wood data from the Polish Basin.

### Carbon isotope ratios and organic matter enrichment

Trends in TOC (Figs 2 and 4; Supplementary Table 1) through the Early Toarcian diverge strongly between West European open marine basins and the marginal-marine Polish basin (e.g. ref. 11, Fig. 2). In open marine basins more negative δ^13^C values are associated with higher TOC content, i.e. the T-OAE is associated with a high rate of organic burial, whereas coeval sediments deposited in the Polish Basin show very low TOC contents. In the Toarcian Polish Basin the highest content of TOC occurs instead in the sediments deposited after the Pliensbachian-Toarcian boundary event and before the onset of the T-OAE (Fig. 2).

Anoxic conditions in the Polish basin occurring before the onset of the T-OAE could offer an explanation of higher earliest Toarcian TOC content^50^. However, throughout the whole Lower Toarcian of Poland, colour and element geochemistry contradict such anoxia. HH-XRF analyses do not indicate enrichments of redox sensitive metals (e.g. Mo, Cu; see Supplementary Table 1) at intervals corresponding to the stepped negative CIE of the Early Toarcian in the investigated core materials. Many redox-sensitive elements have short oceanic residence times (e.g. Co, Cu, Cr), such that the geology of the local hinterland can significantly impact on the delivery of such elements into the basin. Mo, however, has an oceanic residence time on the order of 10^6^ years, so that its concentration in seawater is significantly higher than in river waters and its behaviour in brackish and marine successions – everything else equal – should be similar. Since our record does not indicate any significant enrichment in this element (Supplementary Table 2), we cannot propose any elemental indication for widespread oxygen depletion in the Polish Basin. Framboidal pyrite observed in the interval preceding the T-OAE is too large (diameters significantly exceeding 10 μm) to have formed in an anoxic water column and is therefore likely of diagenetic origin. In a shallow, marginal marine setting the development of a thermal or salinity-driven stratification leading to a (partially) anoxic water column is less likely to have occurred than in more distal settings. Thus the probable absence of anoxic conditions is attributed mostly to the general shallowness of the basin and destruction of the halocline. Considering the geochemical, mineralogical, sedimentological and biological evidence showing no markers of anoxia, the variability in TOC contents in the Polish Late-Pliensbachian to Early Toarcian successions is rather a reflection of the efficiency of terrestrial biodegradation.

Indeed, the Oxygen Index (OI) of the TOC, reflecting the degree of kerogen decomposition (mineralization) points to a high degree of microbial oxygenation/decomposition of terrestrial kerogen in the T-OAE interval, where TOC concentrations are lowest.

Nonlinear mechanisms could involve cumulative sequestrations and subsequent recycling of carbon on astronomical time scales by episodic accumulation and decay of organic carbon in quasi-stable terrestrial reservoirs^51^. Such conspicuous sequestration of carbon occurred in the latest Pliensbachian, particularly just before a transgression at the beginning of Toarcian, when dark, carbon-rich strata were deposited in large parts of the Polish basin^23^ (Fig. 2). As indicated by^52^, it could be linked to the long-term, low-magnitude eccentricity cycle of ~9 mya, which caused disturbances in orbital forcing during the Pliensbachian and produced strong amplitudes in carbon isotope composition. Furthermore, it is possible that the carbon-rich strata formed in the hinterland of the Polish Basin during the latest Pliensbachian could have been partly exposed (due to enhanced hydraulic cycle), and decomposed during the T-OAE, additionally accelerating climate warming.

Fungal spore peaks linked to a relative and absolute loss of wood suggest a prominent role of fungal wood decomposers (saprophytes, the largest group of fungi) on soil dynamics during peak greenhouse conditions during the T-OAE. Most likely, extreme greenhouse conditions (high temperature in particular), created favourable conditions for detrittivore fungi, the only organisms which were able to degrade lignin tissues^29^, building the most common and recalcitrant terrestrial maceral – wood. Similar processes leading to decomposition of lignin in soil are observed in recent experiments involving soil warming^4^. Furthermore, enhanced humidity and elevated pCO_2_ during the T-OAE would have assisted enhanced soil organic matter decomposition (2,3). The co-variation of humidity-sensitive plant abundance (Equisetaceae, bryophyte and lycopsids) and fungal spores counts (Fig. 2) is weaker than the correlation between the frequency of fungal spores and values of carbon isotopes (tracing temperature changes). Humidity, though sufficiently high for fungal development throughout the whole T-OAE interval, is therefore thought to have been of a lesser significance for dynamics of fungal decomposition than elevated temperature. As marine O_2_ respiration in shallow marine basins seems to be of lesser significance^53^, the kerogen studied herein had likely been oxygenated on land prior to burial and before delivery to the receiving basin.

**Figure 3:**
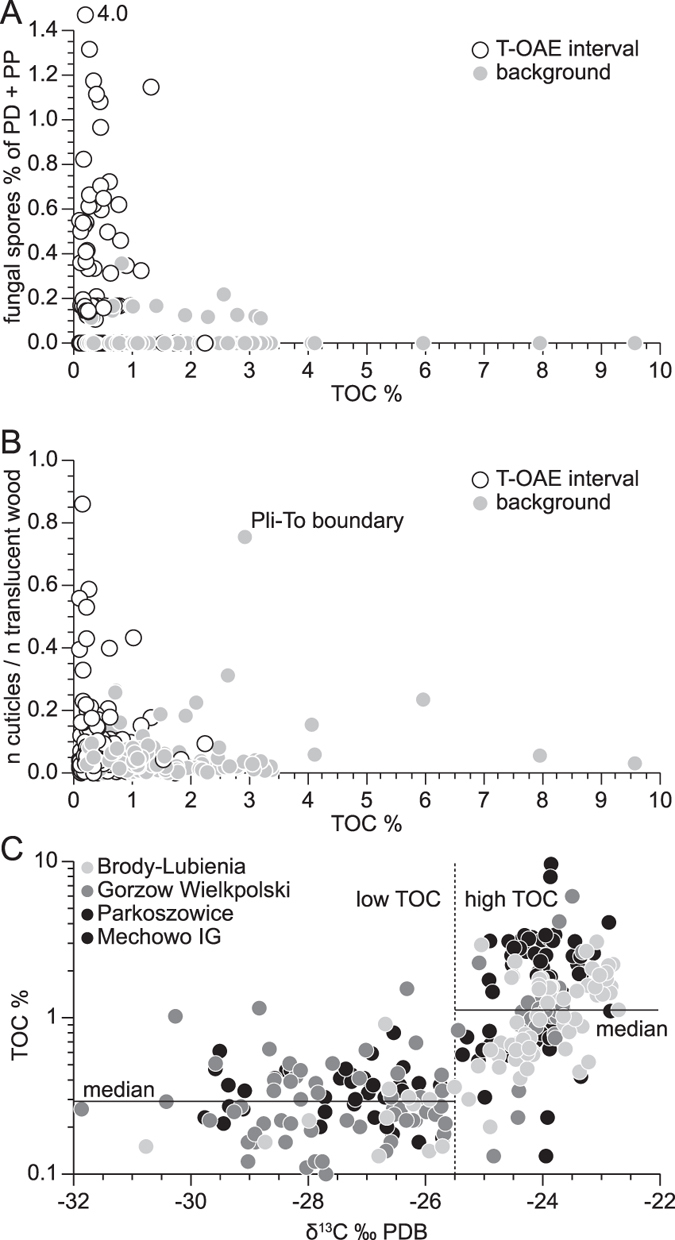
Cross plots of climate-sensitive indicators in the Lower Toarcian strata of Poland in all four boreholes ( Fig. 2). (**A**) Ratio of fungal spore counts with the sum of Palinodebris (PD) and Palinomorphs proper (PP) plotted against TOC. Samples representing the T-OAE interval: white circles; non-T-OAE samples: grey circles. (**B**) The cuticle/translucent wood ratio vs TOC; note one exceptional non-T-OAE sample representing the Pliensbachian-Toarcian boundary event (Pli-To boundary). (**C**) Plot of δ^13^C versus TOC concentration in the four boreholes. Systematic fluctuation of TOC preservation throughout the T-OAE parallel to variations in δ^13^C generate two populations of data separated at δ^13^C of −25.5‰. Samples with low TOC and low δ^13^C dominate in the T-OAE and samples with high TOC and high δ^13^C in the remainder of the succession.

**Figure 4:**
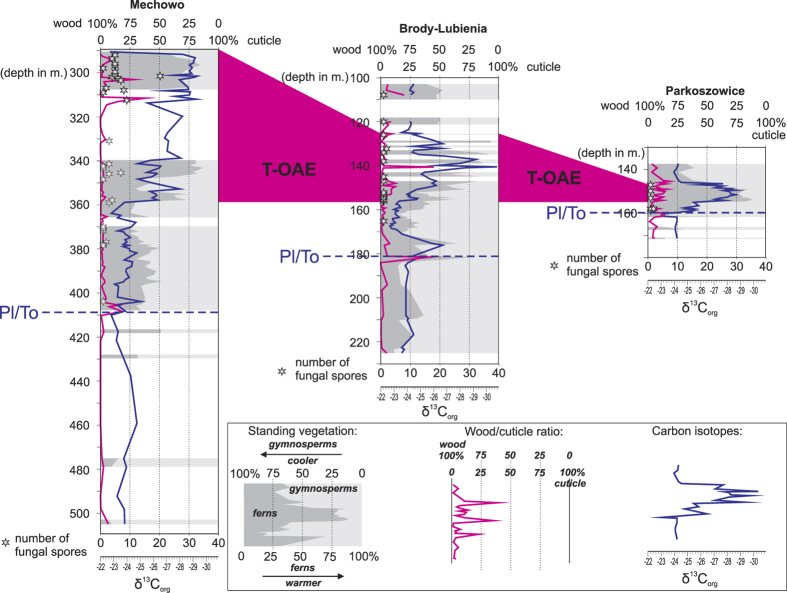
Correlation between climate-sensitive indicators (standing vegetation, wood/cuticle ratio, carbon isotopes and frequency of fungal spores) in three boreholes. Note that climate warming interpreted from palynomorph frequency and carbon isotope curves acted in concert with presence or higher frequency of fungal spores, which is reflected in wood decomposition and enhanced cuticular sequestration. Similar, although less marked and short-lasting effect can be observed at the Pliensbachian-Toarcian boundary warming event. Blank fields mean scarce or lack of palynomorph data, in Brody-Lubienia borehole the interval 110–120 m was missing in the core record.

**Figure 5:**
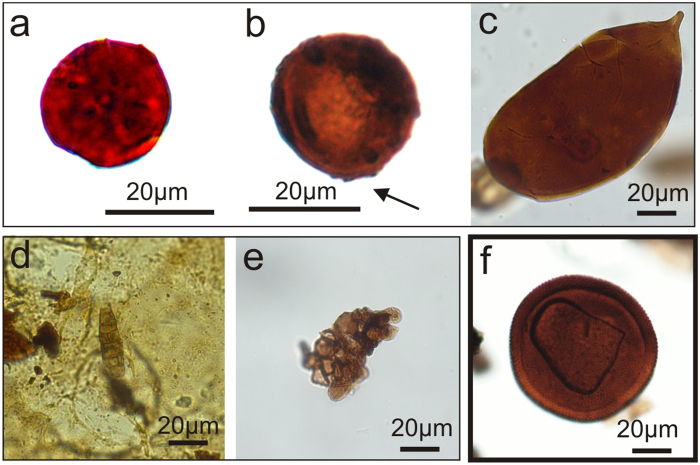
Fungal spores (**a–e**): a – sphaerical, unicellular amerospore – note punctuate surface; (**b**) sphaerical, unicellular amerospore, with possible hyphae attachment (arrowed); (**c**) sack-shaped unicellular amerospore; (**d**) phragmospore with transverse septa; (**e**) multicellate dictyospore; (**f**) example of Prasinophyceae (green algae), showing characteristic features (thick wall and scabrate surface), which differ these palynomorphs from fungal spores.

Overall, palynomorph frequency and carbon isotope peaks co-vary with the presence and higher frequency of fungal spores (Figs 4 and 5), which is reflected in wood decomposition and enhanced cuticular sequestration (Figs 3 and 4). Fungal spore peaks linked to a relative and absolute loss of wood (Fig. 4) therefore suggest a prominent role of fungal wood decomposers (saprophytes, the largest group of fungi) on soil dynamics during peak greenhouse conditions.

### The Pl/To boundary, a weak OAE?

The vegetation response to the environmental perturbation occurring at the Pl/To boundary is similar, but weaker than during the T-OAE. Palynomorph response is observed in the Brody-Lubienia core, but correlative intervals in Mechowo and Parkoszowice show very weak or no response. Also fungal spores are rare (Mechowo, Parkoszowice) or absent (Brody-Lubienia, Gorzów Wielkopolski). These observations indicate that the Pliensbachian-Toarcian boundary event was of a lesser significance for terrestrial environments than the T-OAE, probably because of less dramatic temperature increase and/or relatively short time of environmental perturbation. Although a transient decrease in TOC can be observed at the Pl/To transition, it is likely that the Pl/To warming event was not sufficiently severe (as the T-OAE interval) to cause mass decomposition of the terrestrial carbon pool.

### Significance of terrestrial carbon pool for OAE dynamics

An approximate carbon budget can be estimated from the area of the Polish Basin (~320,000 km^2^ Fig. 1), based on comparison between average TOC of the T-OAE interval (c. 0.5% TOC in 20 m) and the pre-T-OAE Toarcian strata (c. 2.0% TOC) in the Mechowo and Brody boreholes, located along the axial zone of the basin and deposited under similar sedimentation rate^7^,^18^,^23^. It follows that in this basin alone c. 100 km^3^ (c. 200 Gt) carbon, equivalent to c. 730 Gt CO_2_, was not sequestered during the T-OAE - in comparison to the “background” pre-T-OAE period. The amount of kerogen (plant litter) decomposed in the hinterland of the Polish Basin must have been by an order of magnitude higher^54^. Moreover, rather than accommodating this loss of organic matter by a slight, sustained increase in decomposition^55^, it is more likely that organic matter was accumulated in the soil zone during moderate climatic conditions and then decomposed in rapid “bursts”. Indeed, elevated TOC contents and less oxygenated organic matter (lower OI) between the CIE peaks, pointing to somewhat higher degree of carbon sequestration, reflect this dynamic soil response (Fig. 2). The terrestrial carbon pool in the mid-high palaeolatitudes would thus have globally acted as a labile reservoir and bursts of soil organic matter decomposition (Fig. 2) would have been very short-lived (perhaps only few hundred years long)^26^,^31^. Due to prevailing hot and arid conditions^14^, the tropical zones where likely less sensitive to this mechanism.

The organic matter composition decomposition - in conjunction with methane outgassing in oceans (~2000–5000 GtC from methane hydrate reservoirs^52^) and thermogenic methane release connected to the intrusion of Karoo-Ferrar dolerites into Gondwanan coals (~2600–4400 GtC–20) might have been one of the warming triggers leading up to peak CIEs^51^,^52^. If soils in other regions behaved similar to the Polish hinterland during the T-OAE, and accounted for a similar amount of organic matter as in modern times^5^,^9^, the rapid mobilization of this reservoir would have had the potential to contribute to a negative CIE even in a high pCO_2_ world^20^,^55^. An assumed atmospheric pCO_2_ of ~4 times pre-industrial levels would translate into an atmospheric and surface ocean carbon reservoir of ~5000 Gt C during the Toarcian. A fast release of organic matter corresponding to half the modern soil carbon pool (c. 700 Gt)^9^ under such conditions could contribute to a CIE with >−2‰, if the carbon dioxide was released too fast to be incorporated into the deep ocean carbon reservoir efficiently. As suggested by ref. 42, the corrected 3–4‰ magnitude of the T-OAE carbon cycle perturbation implies the injection of lower amounts of carbon than previously modelled, making the contribution of soil organic matter potentially more significant. Further studies on global terrestrial response to the Toarcian OAE, however, are necessary to elucidate, whether the situation as observed in the Polish Basin are truly representative.

## Conclusions

Up to ~7‰ δ^13^C negative excursions in terrestrial organic matter (separated wood), reflect the change in carbon-isotope composition of atmospheric CO_2_ rather than organic matter mixing or enhanced δ^13^C fractionation in plants due to varying regional humidity. The positive feedback loop of increased temperature and humidity, facilitating the decomposition of plant litter and further release of CO_2_ and CH_4_ into the atmosphere, may have played a major role in observed aggravation of the Toarcian greenhouse climate during the T-OAE.

The frequency of fungal spores, which is correlated with negative C isotope peaks and enhanced cuticular plant litter sequestration, pointing to climate-driven enhanced decomposition of wood and rapid destruction of terrestrial carbon pool, which in turn may have played an important role in the aggravation of the Jurassic greenhouse disaster.

Our results from geological past extend the evidence that climate warming has the potential to reduce terrestrial carbon storage, particularly the largest terrestrial carbon pool – soil. Crossing a climatic threshold, this carbon reservoir is affected by soil organic matter decomposition (respiration) and becomes an additional source of greenhouse gasses, amplifying greenhouse-driven environmental change. These findings advance our understanding of future climate warming dynamics and inform the debate about anthropogenic environmental change.

## Methods

Four well-preserved and continuous cores were studied (Figs 1 and 2), which yielded lithological, sedimentological and geochemical data previously discussed for the Late Pliensbachian-Early Toarcian interval by refs 18,23,24,25,27,32. These earlier studies of the green, grey, brownish mudstones and grey sandstones of the Ciechocinek Formation are here revisited and appended by palynological, organic matter data, colour and element geochemical data (Fig. 2; Supplementary Table 1).

### Palynology

Organic matter was concentrated using standard palynological techniques applied in the laboratory of the Polish Geological Institute-National Research Institute: 294 samples (about 30–50 g sediment) were crushed and treated twice with cold HCl (30%) and cold HF (38%); the first residues were washed in water with hydrogen iodide acid, then heavy minerals (such as pyrite) were separated using CdJ and KJ salt solution, and finally the residue was washed in distilled water to neutral pH. During the preparation of the organic residues no chemical oxidation, organic reagents, or deliberate physical separation was used. Palynofacies analysis was carried out on 93 samples from Mechowo IG-1, 81 samples from Brody-Lubienia, 42 samples from Parkoszowice and 92 samples from Gorzów Wielkopolski. 600 palynomorphs (terrestial and marine) were counted per slide using a light microscope and data are presented as percentages of total palynomorph assemblage. To receive percentage share of plant groups, about 200 spores and pollen taxa were counted per slide.

### Geochemistry

Hand-held X-ray fluorescence (HH-XRF) analyses of 294 samples from four boreholes were performed at the University of Copenhagen using an InnovX Professional HH-XRF. Sample powders were transferred into plastic beakers with circular openings (17 mm diameter) and the opening covered with LDPE foil (9.5 μm thickness) and fixed with a rubber band. For the measurements the beakers were turned upside down, so that the powdered material created a flat surface on the LDPE foil. Analyses were done in a sequence of two integrations at different excitation energies (40 kV for heavy elements and 10 kV for light elements) with integration times of 120s each. Reproducibility of the analyses was tested by multiple measurements of the silicate reference material PACS-2 (n = 13; Supplementary Table 1). Biases with respect to values published by ref. 56 are −33% for Al (6.37%) and −9.3% for K (1.26%). Trace elements show larger bias, rendering absolute numbers unreliable, but generally reproduce within 10% (2 rsd) in the concentration range ≥10 μg/g, robustly showing relative variations of concentrations throughout the analyzed successions.

### Rock Eval 6 pyrolysis

In total, splits from 88 (100–150 g) carbonate-free rock samples from two boreholes (Mechowo IG-1, Brody-Lubienia) were studied in the Rock Eval pyrolysis apparatus - model 6 Turbo (Vinci Technologies), in the laboratory of the Polish Geological Institute (Supplementary Table 1). Crushed bulk rock material was thermally decomposed in a helium or nitrogen atmosphere. Every sample was heated to 650°C. Repeat analyses of the parameters S1 (thermally liberated free hydrocarbons), S2 (volatile components released during pyrolysis), S3 (CO2 generated from kerogen) and TOC agree within ±0.05. The Oxygen Index (OI, in mg CO2/g TOC) is calculated according to the formula (S3*100)/TOC. Hydrogen index (HI) = S2 (mg/g)/%TOCx100.

### Total Organic Carbon (TOC)

Analyses of 287 samples were performed using the chromatographic, coulometric method (procedure PB –22) using an automated LECO analyser. Minor differences between LECO and Rock Eval 6 TOC in 25% of measurements were found, and for consistency only the LECO results which provide higher-resolution information are presented in the figures (Supplementary Table 1; Fig. 2).

### Carbon isotopes

δ^13^C values from >400 analyses of woody phytoclasts (xylem) separated manually from palynological preparations are taken from ref. 18 and the analytical method is described therein.

All materials (palynological preparates and rock samples) are stored in the Polish Geological Institute, Warsaw.

## Additional Information

**How to cite this article**: Pieńkowski, G. *et al*. Fungal decomposition of terrestrial organic matter accelerated Early Jurassic climate warming. *Sci. Rep.* **6**, 31930; doi: 10.1038/srep31930 (2016).

## Supplementary Material

Supplementary Information

Supplementary Information
